# The Outlook of the Profession to the Young M. D.

**Published:** 1893-08

**Authors:** Peel M. Payne

**Affiliations:** Terrell


For Texas Medical Journal.

### THE OUTLOOK OF THE PROFESSION TO THE YOUNG M. D.

BY PEEL M. PAYNE, M. D., TERRELL.

(Read before the Terrell Medical Society, and voted to TEXAS MEDICAL JOURNAL.)

In identifying myself with the Terrell Medical Society and accepting the duty assigned of writing a paper, it is in the hope of being benefited by the counsel and wisdom of the older members, rather than a desire to impart instruction or offer anything new.

It is with a feeling of diffidence, I suppose common to young men just entering on a professional career, that I attempt to write.

But viewing the profession that I have just espoused from a moral, financial and purely intellectual standpoint, I think I can see many difficulties, any of which may forever obstruct the young man in his way to success.

Financially, I do not see any rich doctors; morally, I do not know that they are any better than preachers, but I believe that the medical profession offers an intellectual field, which calls into play the higher intellectual operations, not only in the study of human nature, as exemplified by the beggar in his hovel, or the rich man in his palatial mansion, but in tracing the relations of cause and effect, or in studying the uniformity of nature's laws, whether in the law of "multiple proportion," "proximate principles;" or the action of a nerve cell; or the construction of the eye, in obedience to the law of optics, in focussing parallel rays of light on the retina; or the wonderful mechanism of the human heart as a blood pump, forcing the vitalized fluid to every cell of the body, furnishing its particular pabulum whether it be bone, muscle or nerve cell, to say nothing of pathological processes which are simply a disturbance of nature's laws.

To the young physician, with this grand scientific panorama rising before his intellectual horizon, and feeling that the germ theory has just revolutionized the entire theory of medicine, which teaches that the human body is simply a battle field where the vital forces and germ life wage the fiercest warfare, with the odds often in favor of the latter, I undertake to say, it is enough to intimidate the mind of a Franklin and a Newton; and to the young M. D., whose ideational cells are just taking shape, it is enough to cause him to go into an intellectual swoon and exclaim, "I know very little, and am not very certain about that."

